# Identification of Novel Diagnostic Biomarkers in Prostate Adenocarcinoma Based on the Stromal-Immune Score and Analysis of the WGCNA and ceRNA Network

**DOI:** 10.1155/2022/1909196

**Published:** 2022-01-15

**Authors:** Tengfei Zhang, Yaxuan Wang, Yiming Dong, Lei Liu, Yikai Han, Huanrong Wang, Qian Wei, Peige Xia, Wang Ma, Lifeng Li

**Affiliations:** ^1^Cancer Center, The First Affiliated Hospital of Zhengzhou University, Zhengzhou 450052, China; ^2^Medical College, Henan Polytechnic University, Jiaozuo 454001, China; ^3^Department of Cardiology, The First Affiliated Hospital of Zhengzhou University, Zhengzhou 450052, China; ^4^Department of Orthopedics, The First Affiliated Hospital of Zhengzhou University, Zhengzhou 450052, China

## Abstract

Prostate cancer is still a significant global health burden in the coming decade. Novel biomarkers for detection and prognosis are needed to improve the survival of distant and advanced stage prostate cancer patients. The tumor microenvironment is an important driving factor for tumor biological functions. To investigate RNA prognostic biomarkers for prostate cancer in the tumor microenvironment, we obtained relevant data from The Cancer Genome Atlas (TCGA) database. We used the bioinformatics tools Estimation of Stromal and Immune cells in Malignant Tumor tissues using Expression data (ESTIMATE) algorithm and weighted coexpression network analysis (WGCNA) to construct tumor microenvironment stromal-immune score-based competitive endogenous RNA (ceRNA) networks. Then, the Cox regression model was performed to screen RNAs associated with prostate cancer survival. The differentially expressed gene profile in tumor stroma was significantly enriched in microenvironment functions, like immune response, cancer-related pathways, and cell adhesion-related pathways. Based on these differentially expressed genes, we constructed three ceRNA networks with 152 RNAs associated with the prostate cancer tumor microenvironment. Cox regression analysis screened 31 RNAs as the potential prognostic biomarkers for prostate cancer. The most interesting 8 prognostic biomarkers for prostate cancer included lncRNA LINC01082, miRNA hsa-miR-133a-3p, and genes TTLL12, PTGDS, GAS6, CYP27A1, PKP3, and ZG16B. In this systematic study for ceRNA networks in the tumor environment, we screened out potential biomarkers to predict prognosis for prostate cancer. Our findings might apply a valuable tool to improve prostate cancer clinical management and the new target for mechanism study and therapy.

## 1. Introduction

Prostate cancer (PRAD) is one of the most common types of cancers and the third leading cause of death from cancer in men worldwide [1, 2]. The number of new prostate cancer cases globally in 2030 is estimated to be 1.7 million [2]. Prostate cancer is still a significant global health burden in the coming decade. Surgery, chemotherapy, radiation therapy, hormone therapy, and immunotherapy such as Sipuleucel-T and PD-1/PD-L1 immune checkpoint inhibitors are the available treatment for prostate cancer patients nowadays. Although the five-year survival is optimistic for most of the early stage, the survival for a distant and advanced stage of prostate cancer still needs improvement [3]. The therapeutic resistant and metastatic progression of cancer cells was significantly associated with their surrounding tumor microenvironment [4, 5]. The tumor microenvironment consists of cancer cells, stromal cells (including endothelial cells, fibroblasts, and various types of immune cells) and the extracellular matrix (ECM) produced by stromal cells [5]. The crosstalk between cancer cells, stromal cells, and immune cells is the major driver for the biological functions of tumors. Prostate cancer is the solid malignancy having a highly immunosuppressive microenvironment [6, 7]. Therefore, the molecular events associated with the dynamic regulation in tumor microenvironment are valuable for screening prognosis biomarkers for prostate cancer.

Competitive endogenous RNA (ceRNA) means the RNA in the complex transcriptional regulation network, including messenger RNAs (mRNA), transcribed pseudogenes, circular RNAs, and long-chain noncoding RNAs (lncRNA). The ceRNA hypothesis suggested that the mutual regulation of RNAs is achieved by competing for microRNA (miRNA) response elements (MRE) of miRNAs [8]. lncRNAs can bind to miRNAs by MRE and prevent the regulatory function of miRNA in mRNA. ceRNA/miRNA axis posttranscriptional regulation is one of the current hot topics in cancer research. The crosstalk in the ceRNA network regulates essential biological processes in cancer, indicating the possibilities of ceRNAs as diagnostic and prognosis biomarkers for cancers [9, 10].

Weighted gene co‐expression network analysis (WGNA) is an analysis method for exploring co-expressed gene modules, which can discover the relationship between gene networks and phenotypes of interest, and focus on core genes in the networks [11, 12]. WGCNA can identify highly related genes and cluster them into the same module and provide clinical characteristics of related modules [13], which is very helpful in identifying the candidate biomarkers and a commonly used method in tumor-related research including liver hepatocellular carcinoma [14], breast cancer [15], and lung cancer [16]. However, there is no report yet on the application of WGCNA to find the biomarkers of PRAD.

Our study was aimed at investigating microenvironment-related prognostic biomarkers for prostate cancer targeting the ceRNA network using The Cancer Genome Atlas (TCGA) database. We employed the bioinformatics tools Estimation of Stromal and Immune cells in Malignant Tumor tissues using Expression data (ESTIMATE) [17] and WGCNA [11] to construct a ceRNA network based on the stromal-immune score and screen prognosis lncRNA, mRNA, and miRNA biomarkers for prostate cancer.

## 2. Materials and Methods

### 2.1. Data Acquisition

The genetic test data (RNA-seqv2) were downloaded from The Cancer Genome Atlas (TCGA, https://portal.gdc.cancer.gov, accessed on 15 May 2020) and organized into standardized raw data for subsequent analysis. We excluded samples diagnosed with other cancers and prostate cancer samples missing any lncRNA, miRNA, or mRNA data. Finally, 462 prostate cancer cases were included for further analysis.

### 2.2. Tumor Stromal-Related Differentially Expressed Gene Profile Based on the ESTIMATE Algorithm

ESTIMATE can predict tumor purity and tumor microenvironment (whether it is infiltrated by stromal cells and immune cells) using gene expression data [17]. Briefly, ESTIMATE generates three scores: (1) the stromal score (SS) reflects the level of stroma content in tumor tissue, (2) the immune score (IS) represents the infiltration of immune cells in tumor tissue, and (3) the estimate score infers tumor purity. SS and IS for each of the prostate cancer samples were derived by the R language “estimate” package. Samples are divided into 4 groups according to SI and SS, and the median value is the cut-off value. The differentially expressed mRNA, lncRNA, and miRNA in the SS-high group compared to the SS-low group and the IS-high group compared to the IS-low group were analyzed. *P* value < 0.05, false discovery rate < 0.05, and log2 fold change > 1.2 were considered to indicate significance.

### 2.3. WGCNA

The “WGCNA” package in the R language was used for WGCNA analysis, as described previously [11]. We constructed the lncRNA/mRNA coexpression network and miRNA coexpression network separately. We firstly got Pearson's correlation matrices cor (*i*, *j*) to indicate the correlation of genes and then calculated the weighted adjacency matrix as follows: *a_ij_* = (0.5 × (1 + cor (*i*, *j*))*β*. *a*_*ij*_ refers to the correlation between genes *i* and *j*. *β* as a soft thresholding parameter strengthens the strong correlation while weakening the weak correlation and the negative correlation, making the correlation value more in line with the characteristics of the scale-free network, and has more biological significance [18]. Based on these *β* values, we constructed a topological overlap matrix (TOM) and a hierarchical clustering tree between genes for module detection. Then key coexpression modules were further screened by the number of coexpressed genes, the clinical characteristic correlation analysis, and biological function analysis. It should be noted that in our study, we performed WGCNA preanalysis on the data obtained by SS and IS and finally select the SS group as the data for subsequent analysis.

### 2.4. Correlation Analysis between Coexpression Modules and Clinical Characteristics

Module eigengene (ME) was the first principal component of a given module, which can represent the gene expression profile of the entire module [11, 19]. After we got MEs, the Pearson correlation test was performed to analyze the association between the coexpression gene module and the clinical characteristics. The clinical characteristics included in the analysis have age, Gleason score, stroma score, T stage, N stage, tumor grade, and survival. A significant correlation was considered when *P* value was < 0.05.

### 2.5. Potential Molecular Mechanism and Pathway Analysis

To investigate the molecular mechanisms, we performed Gene Ontology (GO) and Kyoto Encyclopedia of Genes and Genome (KEGG) functional enrichment analysis, applying the Visualization and Integrated Discovery (DAVID). We got multiple results, using the criterion of *P* < 0.05 to get the target pathways.

### 2.6. ceRNA Network Construction

We selected three lncRNA/mRNA modules (turquoise, blue, and brown) and two miRNA modules (turquoise and blue) to construct a ceRNA network. Firstly, we used miRanda (http://www.microrna.org/), TargetScan (http://www.targetscan.org/), miRWalk (http://129.206.7.150/), and PITA (https://genie.weizmann.ac.il/pubs/mir07/mir07_exe.html) to predict target genes of miRNA-mRNA and miRNA-lncRNA in a specific gene module. Then, based on this predicted miRNA-mRNA and lncRNA-miRNA pair, we used Cytoscape 3.7.0 software to construct a ceRNA (lncRNA-miRNA-mRNA) regulatory network.

### 2.7. Survival Analysis

High-throughput genetic testing data for prostate cancer with Relapse-Free Survival (RFS) information from TCGA database was selected. Based on the mRNA, lncRNA, and miRNA expression data of the sample, using R software for a COX single-factor regression model, we constructed the corresponding Kaplan-Meier curves. We carried out RFS survival analysis on the mRNA, lncRNA, and miRNA in the module we selected and found mRNA, which has a significant impact on the survival of RFS lncRNA and miRNA. The *P* value set in the analysis results is 0.05.

## 3. Results

### 3.1. Differentially Expressed Gene Profile in Tumor Stroma

**Figure 1:**
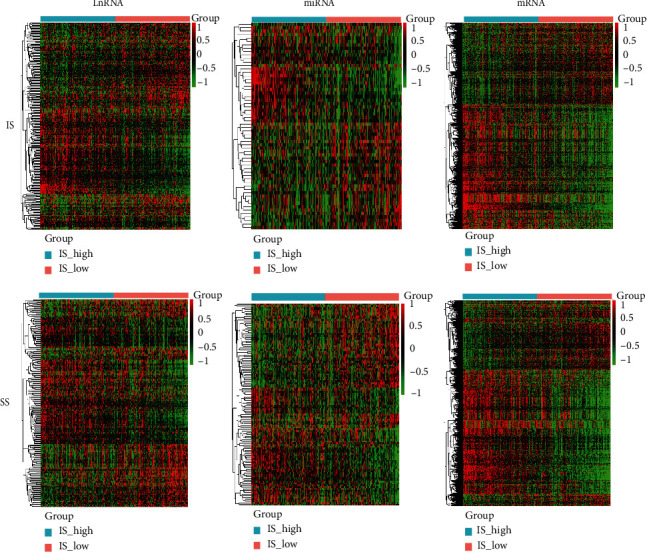
Heatmap of the differentially expressed genes. Heatmap shows the differentially expressed genes of high and low IS and SS in TCGA prostate cancer patient cohort. IS: immune score; SS: stroma score; TCGA: The Cancer Genome Atlas.

A total of 462 tumor samples were used for further analysis, which we get from TCGA database. As shown in Figure 1, we found 781 mRNA, 237 lncRNA, and 60 miRNA differentially expressed in the IS-high group compared to the IS-low group and 765 mRNA, 207 lncRNA, and 116 miRNA differentially expressed in the SS-high group compared to the SS-low group. The significant functions of the GO and KEGG signaling pathway were screened (Table 1). The differentially expressed genes from comparison of both IS and SS were significantly enriched in immune response. The metabolism pathway, PI3k-AKT signaling pathway, cancer-related pathways, and cell adhesion-related pathways were the top pathways in differentially expressed genes from comparison of both IS and SS.

### 3.2. Construction of the Weighted Coexpression Network

After the above analysis, the differentially expressed genes of IS and SS were obtained, and then, WGCNA preanalysis was performed, respectively. However, no meaningful results were obtained in the IS group and the subsequent analysis could not be performed, so we selected the SS group as the research data.

**Figure 2:**
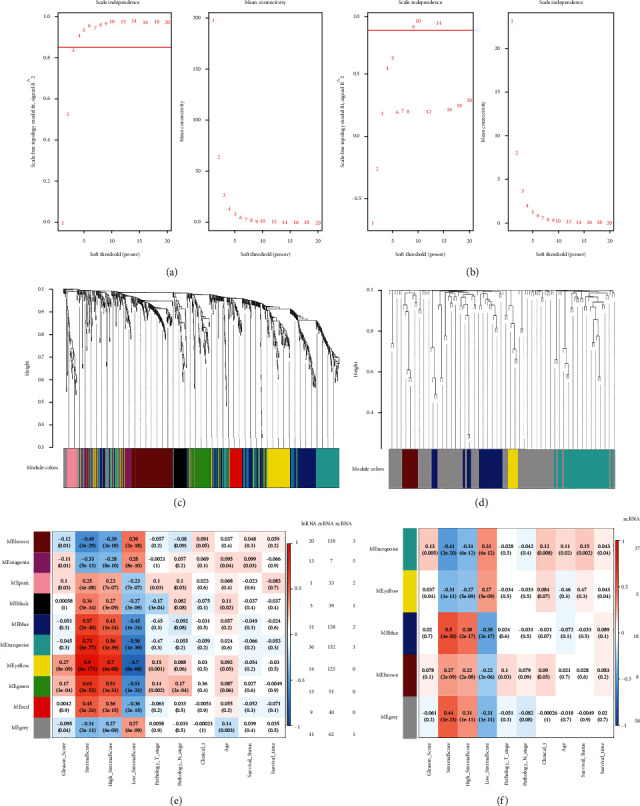
Weighted coexpression network-related graphics. (a, b) Determine the soft thresholding parameter in the lncRNA/mRNA (a) and the miRNA (b) WGCNA. The scale-free fit index and mean connectivity of various soft threshold parameters (*β*) are shown on the left and right picture, respectively. (c, d) Cluster dendrogram of lncRNA/mRNA (c) and miRNA (d) coexpression modules identified by *β* value. Colors are associated with coexpression modules (color gray represents no lncRNA/mRNA or miRNA assigned). (e, f) The association between different lncRNA and mRNA modules (e), miRNA modules (f), and the clinical characteristics of prostate cancer patients. The numbers listed on the right to the heatmap are the number of lncRNA, mRNA, and ncRNA in the lncRNA and mRNA module or the number of miRNAs in the miRNA module. lncRNA: long-chain noncoding RNA; mRNA: messenger RNA; miRNA: microRNA; WGCNA: weighted gene coexpression network analysis; ncRNA: noncoding RNA.

We chose the 765 mRNAs, 207 lncRNAs, and 116 miRNAs differentially expressed in the SS-high group for coexpression network construction. In order to make the connections between genes in the network obey the scale-free network distribution while taking into account the average connectivity, the *β* values we finally chose in the coexpression network analysis of lncRNAs/mRNAs (Figure 2(a)) and miRNAs (Figure 2(b)) were 4 and 9, respectively. Next, the hierarchical clustering tree was obtained through the correlation coefficient between genes (Figures 2(c) and 2(d)).

Then, using Pearson's correlation test analysis method, the correlation between gene modules and clinical phenotypes was calculated, and trait-related modules were identified. According to Figures 2(e) and 2(f), the turquoise miRNA module was significantly associated with Gleason score, tumor clinical stage, and survival; the blue lncRNA and mRNA module was associated with N stage, and the brown lncRNA and mRNA module was associated with Gleason score, N stage, and tumor clinical stage.

Regarding GO functional analysis, we noticed that the significant functions in the turquoise module were signal transduction, cell adhesion, cell shape regulation, and several stromal cell-related functions. For the blue module, mRNAs were significantly enriched in signal transduction, oxidative-reduction process, cell differentiation, and gene expression regulation. The related functions of mRNA in the brown module are mainly related to several metabolic-related processes (Table 2). KEGG pathway analysis showed that the mRNAs in the turquoise module were mainly related to focal adhesion, pathway in cancer, cGMP-PKG, cAMP, and MAPK signaling pathway. In the blue module, the mRNAs were associated with metabolic pathway, tight junction, and focal adhesion. In the brown module, the mRNAs were associated with metabolic pathways and PI3K-Akt signaling pathway.

Combined with the number of differential expressions of lncRNA and miRNA, the correlation between module and traits, the biological function, and signaling pathway analysis, we chose turquoise, blue, and brown lncRNA and mRNA modules and turquoise and blue miRNA modules as the key modules for the next ceRNA network analysis.

### 3.3. Module-ceRNA Analysis

**Figure 3:**
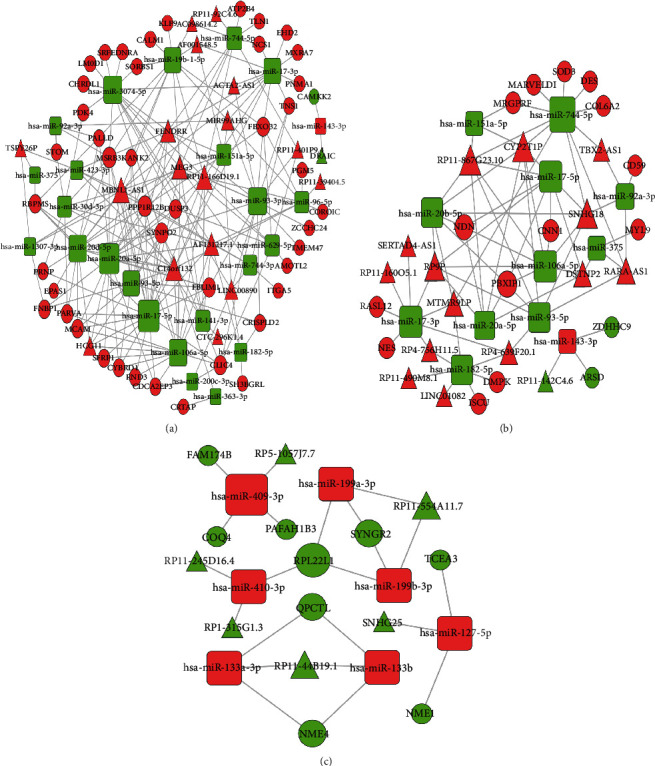
ceRNA networks constructed in the tumor microenvironment in prostate cancer. (a) Turquoise mRNA and lncRNA module and turquoise miRNA module. (b) Blue mRNA and lncRNA module and turquoise miRNA module. (c) Brown mRNA and lncRNA module and blue miRNA module. The shape represents RNA type: rectangle—miRNA, circle—mRNA, and triangle—lncRNA; the color represents the gene expression trend: red—upregulation and green—downregulation; the size of the shape represents the intensity of regulation between RNAs. ceRNA: competitive endogenous RNA; mRNA: messenger RNA; lncRNA: long-chain noncoding RNA; miRNA: microRNA.

Based on the key modules we identified above, we have three combinations for ceRNA network analysis: group 1: turquoise mRNA and lncRNA module and turquoise miRNA module, group 2: blue mRNA and lncRNA module and turquoise miRNA module, and group 3: brown mRNA and lncRNA module and blue miRNA module. Then, we used the predictive miRNA-mRNA and miRNA-lncRNA pair to build the internally competitive ceRNA network (Figure 3). The network for group 1 includes 45 mRNAs, 18 lncRNAs, and 24 miRNAs; that for group 2 includes 16 mRNAs, 15 lncRNAs, and 12 miRNAs; and that for group 3 had 9 mRNAs, 6 lncRNAs, and 7 miRNAs.

### 3.4. RFS Survival Analysis

**Figure 4:**
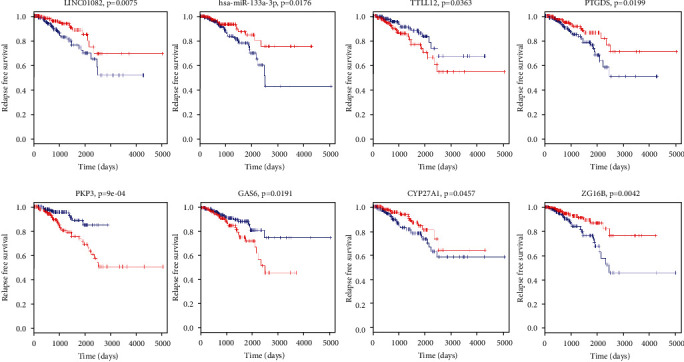
KM survival curves. KM curves of prognostic biomarkers lncRNA LINC01082, miRNA hsa-miR-133a-3p, and genes TTLL12, PTGDS, GAS6, CYP27A1, PKP3, and ZG16B. Abbreviation: KM: Kaplan-Meier; lncRNA: long-chain noncoding RNA; miRNA: microRNA.

RFS survival analysis was performed on all the mRNA, lncRNA, and miRNA found in the above three ceRNA network by constructing the univariate Cox proportional hazards regression model (*P* < 0.05). We found eight lncRNAs, three miRNAs, and twenty mRNAs significantly associated with RFS (Table 3). Finally, survival analysis, biological function analysis, and literature researched identified 8 key biomarkers (lncRNA LINC01082, miRNA hsa-miR-133a-3p, mRNA TTLL12, PTGDS, GAS6, CYP27A1, PKP3, and ZG16B) to predict prostate cancer prognosis (Figure 4). The high expression of LINC01082, hsa-miR-133a-3p, PTGDS, CYP27A1, and ZG16B and the low expression of TTLL12, PKP3, and GAS6 were relevant to better prognosis.

## 4. Discussions

The importance of having tumor microenvironment factors to predict the therapy and prognosis has been strengthened due to their essential role in tumor development. This study investigated prostate cancer prognosis biomarkers based on stromal-immune score-based ceRNA network in the tumor microenvironment using bioinformatics tools ESTIMATE and WCGA. Finally, we screened out a panel of 8 RNAs as the potential prognosis biomarkers for prostate cancer. As far as we know, this is the first study systematically that investigated the ceRNA network in the tumor environment in prostate cancer.

Most of the tumor microenvironment and immune scores are based on the Immunohistochemistry (IHC) and hematoxylin-eosin (HE) staining from Formalin-Fixed and Paraffin-Embedded (FFPE) tissue slides, such as microenvironment cell population counter [19], Glasgow microenvironment score [20], and tumor microenvironment of metastasis score [21]. Generally, TCGA samples are not appropriate to evaluate the microenvironment factors. However, due to the development of the bioinformatics tool ESTIMATE, we can empirically quantitate the stromal and immune cells in tumor samples using gene expression data from whole tumor tissue [17]. The ESTIMATE method enormously expands the database used for microenvironment biomarker screening. In addition, the immune score and stromal score combined with their genomic fingerprint can be used to identify tumor microenvironment stromal cells and characterize cancer immunologic landscape [22]. In our study, the ESTIMATE method was used to evaluate differently expressed genes in prostate cancer microenvironment. The enriched biological function and pathways, immune response, PI3k-AKT, cancer pathway, and cell adhesion-related pathway found in differentially expressed genes from ESTIMATE analysis are significant roles in tumor microenvironment. These findings supported our next ceRNA network construction, and prognosis biomarker screening was based on the prostate cancer tumor microenvironment.

A number of studies have shown that the ceRNA network is related to the occurrence and development of prostate cancer. For example, NEAT1 can regulate the epigenetics of target gene promoters to play the role of oncogenes, by increasing ACSL4 via sponging miR-34a-5p and miR-204-5p, or HMGA2 via sponging miR-98-5p, and was found significantly associated with prostate cancer prognosis [23, 24]. PCAT1 promotes prostate cancer proliferation through c-MYC via sponging miR-3667-3p and FSCN1 via sponging miR-145-5p [25]. To conduct our research, the lncRNA-miRNA-mRNA axis was systematically screened using WGCNA bioinformatics tool. WGCNA has the advantage of finding coexpressed gene modules and probing the relationship between each element and clinical characteristics. WGCNA is valuable for investigating candidate biomarkers and has been widely used in several cancers [14].

We constructed three ceRNA modules, including 153 RNAs associated with the prostate cancer tumor microenvironment. From them, we screened out 31 RNAs significantly associated with RFS survival. lncRNA LINC01082, miRNA hsa-miR-133a-3p, and genes TTLL12, PTGDS, GAS6, CYP27A1, PKP3, and ZG16B are the top RNAs having the potential to predict prognosis for prostate cancer. LINC01082 has been found with the potential to predict the prognosis of urothelial bladder carcinoma and colon adenocarcinoma [26, 27]. In our ceRNA network, LINC01082 regulated miR-182-5P. miR-182-5P is related to the occurrence of PRAD and has the potential to predict its diagnosis and metastasis. For prostate cancer sufferers after radical prostatectomy, hsa-miR-133a-3p was found as a new prognostic biomarker [28]. We found that miR-133a-3p constructed a network with miR-133b, lncRNA RP11-44B10.1, and gene QPCTL and NME4. TTLL12, a serum autoantibody, was overexpressed in prostate cancer patients and regulate cytoskeleton, tubulin modification, and chromosome number stability in prostate cancer [29]. PTGDS was downexpressed and has the potential to predict biochemical relapse in prostate cancer [30]. Its biomarker potential for prostate cancer was also found in a proteomic analysis [31]. GAS6 was one of the genes in an early-stage prostate cancer diagnosis model [32]. GAS6 can promote prostate cancer survival by cell cycle arrest and apoptosis inhibition [33]. CYP27A1 was one of the vitamin D pathway genes. It has a certain potential for predicting the prognosis of PRAD patients [34] and shows some effects on prostate cancer chemoprevention based on vitamin D metabolism. The transcription level of CYP27A1 is positively correlated with disease-free survival and negatively correlated with tumor grade [35]. PKP3 is related to the carcinogenicity and aggressiveness of prostate cancer [36]. This result is consistent with our findings that high expression of PKP3 was associated with a worse prognosis. PKP3 plays some roles in the tumor microenvironment, such as regulating cell invasion and tumor formation via MMP7 proteins [37] and regulating adhering junctions and mesenchymal-epithelial transitions by interaction with desmoglein and desmocollin [38]. ZG16B can regulate the Wnt/*β*-catenin pathway and enhance the immunosuppressive activity of myeloid-derived suppressor cells in the tumor microenvironment [39]. Moreover, ZG16B was found as a potential predictor of prostate cancer biochemical recurrence [40]. The above eight lncRNAs, microRNAs, and genes have the biological function in PRAD development and its microenvironment. Our research showed that they have the potential to predict prostate cancer diagnosis and prognosis of PRAD. Regarding other RNAs, their research in prostate cancer is limited, which provides new research ideas and directions for the carcinogenesis and prognosis of prostate cancer.

## 5. Conclusions

We constructed ceRNA networks in the prostate cancer microenvironment and identified lncRNA LINC01082, miRNA hsa-miR-133a-3p, and genes TTLL12, PTGDS, GAS6, CYP27A1, PKP3, and ZG16B as the potential biomarkers to predict prognosis for prostate cancer. Our findings might apply a valuable tool to improve prostate cancer clinical management and the new target for mechanism study and therapy.

## Figures and Tables

**Table 1:** GO function and KEGG pathway analysis for differently expressed genes in the IS high group vs. IS low group and SS high group vs. SS low group.

Group	GO		KEGG	
	Biology function	*P* value	Pathway	*P* value
IS	Signal transduction	5.59*E* − 18	Metabolic pathways	3.56*E* − 10
	Neutrophil degranulation	1.27*E* − 28	Focal adhesion	1.17*E* − 23
	Immune response	4.18*E* − 37	Pathways in cancer	2.07*E* − 10
	Innate immune response	8.04*E* − 28	PI3K-Akt signaling pathway	3.82*E* − 14
	Oxidation-reduction process	5.28*E* − 14	Cell adhesion molecules (CAMs)	7.89*E* − 16

SS	Signal transduction	1.14*E* − 37	Metabolic pathways	1.49*E* − 17
	Proteolysis	2.71*E* − 27	Focal adhesion	4.48*E* − 33
	Positive regulation of transcription from RNA polymerase II promoter	0.001	PI3K-Akt signaling pathway	1.46*E* − 17
	Immune response	1.26*E* − 30	Pathways in cancer	1.89*E* − 13
	Cell adhesion	8.52*E* − 29	ECM-receptor interaction	1.56*E* − 26

IS: immune score; SS: stroma score; GO: Gene Ontology; KEGG: Kyoto Encyclopedia of Genes and Genome.

**Table 2:** GO function and KEGG pathway analysis for the weighted coexpression mRNA network.

Module	GO		KEGG	
	Biology function	*P* value	Pathway	*P* value
Turquoise	Signal transduction	3.22*E* − 04	Focal adhesion	1.31*E* − 11
	Muscle contraction	2.72*E* − 09	cGMP-PKG signaling pathway	1.37*E* − 04
	Cell adhesion	5.68*E* − 04	Pathways in cancer	7.71*E* − 03
	Negative regulation of transcription from RNA polymerase II promoter	2.94*E* − 02	cAMP signaling pathway	3.16*E* − 03
	Regulation of cell shape	4.13*E* − 05	MAPK signaling pathway	5.60*E* − 03

Blue	Signal transduction	7.77*E* − 04	Metabolic pathways	4.15*E* − 04
	Oxidation-reduction process	1.90*E* − 04	Vascular smooth muscle contraction	1.79*E* − 03
	Cell differentiation	1.18*E* − 03	Tight junction	7.28*E* − 03
	Positive regulation of gene expression	2.66*E* − 03	Mineral absorption	1.42*E* − 03
	Muscle contraction	2.68*E* − 04	Focal adhesion	6.23*E* − 02

Brown	Lipid metabolic process	9.51*E* − 03	Metabolic pathways	7.50*E* − 02
	UTP biosynthetic process	1.56*E* − 03	Regulation of actin cytoskeleton	4.14*E* − 03
	CTP biosynthetic process	1.56*E* − 03	PI3K-Akt signaling pathway	9.91*E* − 03
	GTP biosynthetic process	1.56*E* − 03	Proteoglycans in cancer	9.35*E* − 03
	Nucleoside diphosphate phosphorylation	4.78*E* − 03	Pathways in cancer	7.37*E* − 02

GO: Gene Ontology; KEGG: Kyoto Encyclopedia of Genes and Genome; mRNA: messenger RNA.

**Table 3:** Thirty-one RNAs associated with prostate cancer survival.

lncRNA/miRNA	*P* value	Better survival	mRNA	*P* value	Better survival
lncRNA			ARSD	0.0365	High expression
FGD5-AS1	0.0368	Low expression	CNN3	0.0185	Low expression
FRG1HP	0.0383	Low expression	CRISPLD2	0.0164	High expression
GS1-124K5.12	0.0046	Low expression	CYP27A1	0.0457	High expression
LINC01082	0.0075	High expression	GAS6	0.0191	Low expression
RP11-16D22.2	0.0453	High expression	GCAT	0.0359	High expression
RP11-390F4.6	0.0462	High expression	HSPB8	0.0432	High expression
SNHG25	0.0445	Low expression	MT1X	0.0328	High expression
UBXN10-AS1	0.0075	High expression	PGM5	0.0427	High expression
			PKP3	0.0001	Low expression
miRNA			PTGDS	0.0199	High expression
hsa-miR-133a-3p	0.0176	High expression	PVRL2	0.0179	High expression
hsa-miR-133b	0.0135	High expression	RASL12	0.0251	High expression
hsa-miR-379-5p	0.0252	High expression	SEPT7	0.0341	Low expression
			SH3BGRL	0.0109	High expression
			SPOCK3	0.0022	High expression
			SSR4	0.0095	High expression
			TTLL12	0.0363	Low expression
			ULK3	0.0290	Low expression
			ZG16B	0.0042	High expression

lncRNA: long-chain noncoding RNA; miRNA: microRNA; mRNA: messenger RNA.

## Data Availability

The datasets generated and/or analyzed during the current study are available in the following databases: The Cancer Genome Atlas (https://portal.gdc.cancer.gov), miRanda (http://www.microrna.org/), TargetScan (http://www.targetscan.org/), miRWalk (http://129.206.7.150/), and PITA (https://genie.weizmann.ac.il/pubs/mir07/mir07_exe.html).

## References

[1] Bray F., Ferlay J., Soerjomataram I., Siegel R. L., Torre L. A., Jemal A. (2018). Global cancer statistics 2018: GLOBOCAN estimates of incidence and mortality worldwide for 36 cancers in 185 countries. *CA: a Cancer Journal for Clinicians*.

[2] Ferlay J., Shin H. R., Bray F., Forman D., Mathers C., Parkin D. M. (2010). Estimates of worldwide burden of cancer in 2008: GLOBOCAN 2008. *International Journal of Cancer*.

[3] Di Lorenzo G., Buonerba C., Kantoff P. W. (2011). Immunotherapy for the treatment of prostate cancer. *Nature Reviews. Clinical Oncology*.

[4] Zhang Z., Karthaus W. R., Lee Y. S. (2020). Tumor microenvironment-derived NRG1 promotes antiandrogen resistance in prostate cancer. *Cancer Cell*.

[5] Klemm F., Joyce J. A. (2015). Microenvironmental regulation of therapeutic response in cancer. *Trends in Cell Biology*.

[6] Krueger T. E., Thorek D. L. J., Meeker A. K., Isaacs J. T., Brennen W. N. (2019). Tumor-infiltrating mesenchymal stem cells: drivers of the immunosuppressive tumor microenvironment in prostate cancer?. *The Prostate*.

[7] Jansen C. S., Prokhnevska N., Kissick H. T. (2019). The requirement for immune infiltration and organization in the tumor microenvironment for successful immunotherapy in prostate cancer. *Urologic Oncology*.

[8] Salmena L., Poliseno L., Tay Y., Kats L., Pandolfi P. P. (2011). A _ceRNA_ Hypothesis: The Rosetta Stone of a Hidden RNA Language?. *Cell*.

[9] Liu Y., Xue M., du S. (2019). Competitive endogenous RNA is an intrinsic component of EMT regulatory circuits and modulates EMT. *Nature Communications*.

[10] Sumazin P., Yang X., Chiu H. S. (2011). An extensive microRNA-mediated network of RNA-RNA interactions regulates established oncogenic pathways in glioblastoma. *Cell*.

[11] Langfelder P., Horvath S. (2008). WGCNA: an R package for weighted correlation network analysis. *BMC Bioinformatics*.

[12] DiLeo M. V., Strahan G. D., den Bakker M., Hoekenga O. A. (2011). Weighted correlation network analysis (WGCNA) applied to the tomato fruit metabolome. *PLoS One*.

[13] Yao Y., Zhang T., Qi L. (2019). Integrated analysis of co-expression and ceRNA network identifies five lncRNAs as prognostic markers for breast cancer. *Journal of Cellular and Molecular Medicine*.

[14] Bai K. H., He S. Y., Shu L. L. (2020). Identification of cancer stem cell characteristics in liver hepatocellular carcinoma by WGCNA analysis of transcriptome stemness index. *Cancer Medicine*.

[15] Jia R., Zhao H., Jia M. (2020). Identification of co-expression modules and potential biomarkers of breast cancer by WGCNA. *Gene*.

[16] Niemira M., Collin F., Szalkowska A. (2020). Molecular signature of subtypes of non-small-cell lung cancer by large-scale transcriptional profiling: identification of key modules and genes by weighted gene co-expression network analysis (WGCNA). *Cancers (Basel).*.

[17] Yoshihara K., Shahmoradgoli M., Martínez E. (2013). Inferring tumour purity and stromal and immune cell admixture from expression data. *Nature Communications*.

[18] Zhou Z., Cheng Y., Jiang Y. (2018). Ten hub genes associated with progression and prognosis of pancreatic carcinoma identified by co-expression analysis. *International Journal of Biological Sciences*.

[19] Becht E., Giraldo N. A., Lacroix L. (2016). Estimating the population abundance of tissue-infiltrating immune and stromal cell populations using gene expression. *Genome biology.*.

[20] Park J. H., McMillan D. C., Powell A. G. (2015). Evaluation of a tumor microenvironment-based prognostic score in primary operable colorectal cancer. *Clinical cancer research : an official journal of the American Association for Cancer Research.*.

[21] Rohan T. E., Xue X., Lin H. M. (2014). Tumor microenvironment of metastasis and risk of distant metastasis of breast cancer. *Journal of the National Cancer Institute.*.

[22] Chung W., Eum H. H., Lee H. O. (2017). Single-cell RNA-seq enables comprehensive tumour and immune cell profiling in primary breast cancer. *Nature Communications*.

[23] Jiang X., Guo S., Zhang Y. (2020). LncRNA NEAT1 promotes docetaxel resistance in prostate cancer by regulating ACSL4 via sponging miR-34a-5p and miR-204-5p. *Cellular signalling.*.

[24] Guo Z., He C., Yang F., Qin L., Lu X., Wu J. (2019). Long non-coding RNA-NEAT1, a sponge for miR-98-5p, promotes expression of oncogene HMGA2 in prostate cancer. *Bioscience Reports*.

[25] Xu W., Chang J., Du X., Hou J. (2017). Long non-coding RNA PCAT-1 contributes to tumorigenesis by regulating FSCN1 via miR-145-5p in prostate cancer. *Biomedicine & pharmacotherapy = Biomedecine & pharmacotherapie.*.

[26] Ousati Ashtiani Z., Pourmand G., Salami S. A., Ayati M., Tavakkoly-Bazzaz J. (2017). Dysregulated expression of long intergenic non-coding RNAs (LincRNAs) in urothelial bladder carcinoma. *International journal of molecular and cellular medicine.*.

[27] Huang W., Liu Z., Li Y., Liu L., Mai G. (2019). Identification of long noncoding RNAs biomarkers for diagnosis and prognosis in patients with colon adenocarcinoma. *Journal of Cellular Biochemistry*.

[28] Cheng B., He Q., Cheng Y. (2020). A three-gene classifier associated with microRNA-mediated regulation predicts prostate cancer recurrence after radical prostatectomy. *Frontiers in genetics.*.

[29] Massoner P., Lueking A., Goehler H. (2012). Serum-autoantibodies for discovery of prostate cancer specific biomarkers. *The Prostate*.

[30] Thompson V. C., Day T. K., Bianco-Miotto T. (2012). A gene signature identified using a mouse model of androgen receptor-dependent prostate cancer predicts biochemical relapse in human disease. *International Journal of Cancer*.

[31] Davalieva K., Kiprijanovska S., Maleva Kostovska I. (2018). Comparative proteomics analysis of urine reveals down-regulation of acute phase response signaling and LXR/RXR activation pathways in prostate cancer. *Proteomes.*.

[32] Patel P. G., Wessel T., Kawashima A. (2019). A three-gene DNA methylation biomarker accurately classifies early stage prostate cancer. *The Prostate*.

[33] Lee E., Decker A. M., Cackowski F. C. (2016). Growth arrest-specific 6 (GAS6) promotes prostate cancer survival by G1Arrest/S phase delay and inhibition of apoptosis during chemotherapy in bone marrow. *Journal of Cellular Biochemistry*.

[34] Maksymchuk O. V., Kashuba V. I. (2020). Altered expression of cytochrome P450 enzymes involved in metabolism of androgens and vitamin D in the prostate as a risk factor for prostate cancer. *Pharmacological reports : PR.*.

[35] Alfaqih M. A., Nelson E. R., Liu W. (2017). CYP27A1 loss dysregulates cholesterol homeostasis in prostate cancer. *Cancer research.*.

[36] Breuninger S., Reidenbach S., Georg Sauer C. (2010). Desmosomal plakophilins in the prostate and prostatic adenocarcinomas: implications for diagnosis and tumor progression. *The American journal of pathology.*.

[37] Basu S., Thorat R., Dalal S. N. (2015). MMP7 is required to mediate cell invasion and tumor formation upon Plakophilin3 loss. *PLoS One*.

[38] Franke W. W., Rickelt S. (2011). Mesenchymal-epithelial transitions: spontaneous and cumulative syntheses of epithelial marker molecules and their assemblies to novel cell junctions connecting human hematopoietic tumor cells to carcinomatoid tissue structures. *International Journal of Cancer*.

[39] Escudero-Paniagua B., Bartolomé R. A., Rodríguez S. (2020). PAUF/ZG16B promotes colorectal cancer progression through alterations of the mitotic functions and the Wnt/*β*-catenin pathway. *Carcinogenesis*.

[40] Jin H. J., Jung S., DebRoy A. R., Davuluri R. V. (2016). Identification and validation of regulatory SNPs that modulate transcription factor chromatin binding and gene expression in prostate cancer. *Oncotarget*.

